# Sleepless hours: the lived experience of chronic insomnia and anxiety across the US care spectrum

**DOI:** 10.3389/fpubh.2026.1797563

**Published:** 2026-04-24

**Authors:** Noora Haapala, Helen Allvin, Sumira Riaz

**Affiliations:** 1 Orion Pharma, Espoo, Finland; 2 Unboxed Psychology, London, United Kingdom

**Keywords:** anxiety, chronic insomnia, generalized anxiety disorder, patient journey, qualitative interviews

## Abstract

**Background:**

Chronic insomnia is a prevalent condition associated with significant psychosocial and economic burden. When comorbid with Generalized Anxiety Disorder (GAD), symptom severity is intensified resulting in treatment pathways becoming more complex. Despite the impact, insomnia is often normalized, under recognized as a medical condition, and managed through fragmented care systems.

**Methods:**

A mixed-methods study was conducted in the United States to explore the patient journey of chronic insomnia with and without comorbid GAD. Qualitative insights were generated through 29 semi-structured online interviews with patients (*n* = 16), family members (*n* = 5), and healthcare professionals (*n* = 8), supported by secondary data review. Quantitative symptom data were collected using the Insomnia Severity Index (ISI) and Generalized Anxiety Disorder-7 (GAD-7) questionnaires to characterize the sample. A structured three step approach was applied: data immersion, qualitative research, and thematic analysis.

**Results:**

Findings revealed substantial delays in help seeking and diagnosis, often spanning years or decades, driven by symptom normalization, stigma, and reliance on self-management remedies. Diagnosis was experienced as a moment of relief and validation, though expectations were frequently unmet due to brief consultations, limited information, and reliance on over-the-counter (OTC) options. Treatment was characterized by trial-and-error approaches, with healthcare professionals (HCP) acknowledging an overreliance on pharmacological interventions and systemic barriers to the implementation of cognitive behavioral therapy for insomnia (CBT-I). Ongoing management highlighted adaptation to insomnia as a chronic condition, emotional strain on families, and fragmented care across mental health and sleep services. Across all groups, the comorbidity of insomnia and GAD was recognized as exacerbating symptom severity and complicating management.

**Conclusion:**

This study uniquely captures the lived experience of chronic insomnia and comorbid GAD from the perspectives of patients, family members, and healthcare professionals across the US care spectrum. The findings underscore unmet psychological needs, systemic barriers to nonpharmacological interventions, and the absence of integrated, patient centered care models. Addressing these gaps requires improved recognition of insomnia as a medical condition, expansion of access to CBTI, and inclusion of family perspectives in long term management strategies.

## 1 Introduction

Sleep is a fundamental biological process essential for physical health, emotional regulation, and cognitive functioning. Yet, despite its centrality to wellbeing, insomnia remains one of the most prevalent and burdensome sleep disorders worldwide. This paper reports findings from a qualitative patient journey study conducted in the United States, exploring the lived experience of adults with chronic insomnia, with and without comorbid Generalized Anxiety Disorder (GAD). The US context is particularly salient with inconsistent, predominantly insurance-based US healthcare system creating distinctive barriers to diagnosis and access to evidence-based care, including limited coverage for psychological therapies such as CBT-I, high out-of-pocket costs, and variable availability of specialist services across states. These structural features shape the care pathways of people with insomnia in ways that may differ meaningfully from universal healthcare systems studied elsewhere.

### 1.1 Prevalence and definitions of chronic insomnia and GAD

Despite its widespread prevalence, with approximately 30% of adults reporting at least one symptom of insomnia and 6%−10% of the global population experiencing chronic insomnia, the condition is often misunderstood, under-recognized and insufficiently treated (1, 4, 5). The psychosocial burden of insomnia is substantial, with patients frequently delaying help-seeking due to stigma and a belief that insomnia reflects personal weakness, leading to years of self-management attempts and emotional distress (6).

According to both the DSM-5 and the ICSD-3, chronic insomnia is defined as dissatisfaction with sleep quantity or quality (difficulty initiating or maintaining sleep, or early-morning awakening). This results in clinically significant distress or functional impairment, occurring at least three times per week for a duration of at least 3 months (7, 8). It is important to distinguish this from sleep dissatisfaction arising from insufficient opportunity to sleep, which may occur in the context of demanding employment schedules or caregiving responsibilities; insomnia, by definition, occurs in the context of adequate sleep opportunity. In addition, hyperarousal, a state of heightened physiological, cognitive, and emotional activation, is recognized as an important factor underlying insomnia (9). It can manifest as prolonged sleep onset latency, extended periods of wakefulness during the night, and early morning awakening, and is not limited to difficulties falling asleep alone (9, 10).

Anxiety disorders are common, with lifetime prevalence estimates for Generalized Anxiety Disorder (GAD) ranging from approximately 3.7% in the general population, according to DSM-5 criteria (11, 12). GAD is characterized by excessive anxiety and worry that is difficult to control, associated with symptoms including restlessness, fatigue, difficulty concentrating, irritability, muscle tension, and sleep disturbance (7, 12). To meet diagnostic criteria, these symptoms must cause clinically significant distress or functional impairment, must not be attributable to a substance or another medical condition, and must not be better explained by another mental disorder (12).

### 1.2 Comorbid chronic insomnia and GAD

An estimated 60%−70% of patients with GAD report insomnia symptoms; of this 20%−30% are diagnosed with both insomnia and anxiety disorder (1, 12). Research supports a bidirectional relationship between insomnia and anxiety; the presence of one predicts the pathological development and increased symptom severity of the other (13–15).

Physiological hyperarousal is a characteristic feature of GAD (16–18) that can prolong sleep onset, sustain wakefulness during the night, and fuel uncontrollable ruminative thoughts, thereby increasing anxiety and further impairing sleep quality (14). Whereas abnormal neuroplasticity in insomnia may itself drive hyperarousal states that contribute to GAD symptomatology (19–22). This bidirectional amplification has significant clinical implications, as treating one condition in isolation may yield incomplete outcomes (39).

### 1.3 Treatment landscape for chronic insomnia with or without comorbid GAD

CBT-I is recommended as the first-line treatment for chronic insomnia by major clinical bodies (23, 24). It demonstrates robust efficacy across diverse populations, with higher remission rates than pharmacological approaches and sustained benefit beyond treatment completion (25). However, access to high-quality CBT-I is severely limited in practice with barriers including a shortage of trained practitioners, high costs, time commitment required, and limited integration within primary care pathways (24).

Due to the difficulties with access and cost of CBT-I, pharmacological treatment are commonly used (26). Most pharmacological treatments for insomnia are licensed for short-term use only (typically 4 weeks), and long-term management with medication is not evidence-based for the majority of agents (7, 8, 23). Sleep hygiene advice, recommendations regarding sleep-conducive behaviors and environments, is frequently offered to patients reporting insomnia symptoms, and while it may serve a supportive role, it is not an evidence-based treatment for insomnia disorder in its own right; in clinical trials, sleep hygiene is commonly employed as the control condition rather than as the active intervention (27).

Co-morbid chronic insomnia and GAD can complicate therapy: resistance to GAD treatments can be related to insomnia or sleep deprivation (28–33). While SSRIs, commonly used to treat GAD, may have sedative properties, they can also negatively affect sleep architecture and impair restorative sleep, and insomnia is frequently reported as an adverse effect, particularly at treatment initiation (34).

Beyond its clinical definitions, chronic insomnia represents a hidden epidemic in the United States, with profound psychosocial and economic consequences. Estimates suggest that insufficient sleep reduces US economic productivity by hundreds of billions of dollars annually (35) while workplace studies confirm substantial losses in performance and associated costs (36). Moreover, the economic burden extends to healthcare utilization, where effective insomnia treatments demonstrate a clear return on investment when effectively implemented (37). Yet insomnia remains under-recognized in many psychiatric guidelines, leaving a key gap in integrated, patient-centered care.

### 1.4 Rationale for the present study

O'Regan et al. mapped the insomnia patient journey in Europe and Canada in a 2023 issue of *Frontiers in Public Health* (6), documenting substantial burden, delays in help-seeking, and poor recognition of insomnia as a medical condition. The US healthcare context however, characterized by its insurance-based structure, variability in specialist access, and distinct patient-provider dynamics, has not been comparably examined. Furthermore, prior research has tended to focus on either patient or clinician perspectives in isolation, and has rarely incorporated family member viewpoints or examined the comorbidity of insomnia and GAD as a distinct patient population within a journey mapping framework.

This research addresses these gaps by exploring the patient journey of chronic insomnia with and without comorbid GAD in the United States, capturing the perspectives of patients, family members, and healthcare professionals. By triangulating these stakeholder perspectives, we aimed to generate a holistic understanding of the emotional and clinical experience of insomnia, identify unmet needs across the care pathway, and contribute evidence that can inform patient-centered, integrated care models in the US context.

## 2 Methods

This research employed a mixed-methods design, combining quantitative symptom characterization with qualitative, exploratory interviews to provide a comprehensive understanding of the patient journey of chronic insomnia with or without comorbid Generalized Anxiety Disorder (GAD) in the United States. The study was designed to capture the perspectives of multiple stakeholder groups including patients, family members, and healthcare professionals, recognizing that each group occupies a distinct position in the care pathway and contributes unique and complementary insights. Incorporating all groups within a single study allows for triangulation of perspectives, revealing areas of alignment and divergence that a single-stakeholder approach would obscure.

### 2.1 Participants

A total of 29 individuals took part in online, semi-structured interviews (60 min each). Participants represented three stakeholder groups (see Table 1):

- Patients (*n* = 16): Eight patients with chronic insomnia disorder and eight patients with chronic insomnia disorder comorbid with GAD.
- Family members (*n* = 5): Relatives of patients with chronic insomnia, providing perspectives on the wider psychosocial impact of the condition.
- Healthcare professionals (*n* = 8): Two general practitioners, two sleep specialists, two psychologists, and two psychiatrists (see Table 2).

**Table 1:** Characteristics of patients and family members (United States cohort *n* = 21).

Characteristic	Patients (*n* = 16)	Family members (*n* = 5)	Total sample (*n* = 21)
Age years	Range: 23–74	Range: 20–46	20–74
	Mean: ~55 (approximate)	Mean: ~37	
Gender *n*	Female: 11 (69%)	Female 3: (60%)	Female: 14 (67%)
	Male: 5 (31%)	Male: 2 (40%)	Male: 7 (33%)
ISI total score category *n*	Subthreshold: 3 (19%)		
	Moderate: 7 (44%)		
	Severe: 6 (37%)		
Mean ISI score	18.6		
GAD−7 score category *n*	Minimal / no anxiety: 8 (50%)		
	Mild anxiety: 7 (44%)		
	Moderate anxiety: 1 (6%)		
Mean GAD−7 score	3.4		
Use of medication *n*	Prescription medication: 16 (100%)		

**Table 2:** Characteristics of healthcare professionals (HCPs) participating in the study (*n* = 8).

Role/Title	No. of Cl patients in 3 months	No. of GAD patients in 3 months	Primary healthcare setting	Years in current role
Psychiatrist	101–200	101–200	Community / regional hospital	6–10 years
Psychiatrist	201 +	201 +	Community / regional hospital	More than 10 years
General Practitioner (GP)	201 +	201 +	Family medicine office	More than 10 years
General Practitioner (GP)	51–100	51–100	Outpatient	More than 10 years
Sleep doctor	101–200	101–200	Community / regional hospital	More than 10 years
Sleep doctor	51–100	51–100	Community / regional hospital	More than 10 years
Psychologist	26–50	26–50	Private practice outpatient	More than 10 years
Psychologist	26–50	26–50	Community / regional hospital	6–10 years

Role / title & No of CI patients in 3 months & No of GAD patients in 3 months & Primary healthcare setting & Years in current role.

Patients were recruited to reflect a range of illness durations and experiences, ensuring diversity in perspectives across the insomnia patient journey. Family members were included to capture the broader social and emotional consequences of insomnia and GAD, while healthcare professionals provided insights into clinical management, barriers to care, and unmet needs.

### 2.2 Quantitative component

Participants completed two validated self-report questionnaires to characterize symptom burden and complement qualitative insights:

**Insomnia Severity Index (ISI)** **(****2****)**: A 7-item instrument assessing the nature, severity, and impact of insomnia. Items are rated on a 5-point Likert scale, yielding a total score from 0 to 28, with higher scores indicating greater insomnia severity (2).

**Generalized Anxiety Disorder-7 (GAD-7)** **(****3****)**: A 7-item scale measuring the severity of generalized anxiety symptoms. Items are scored from 0 to 3, with total scores ranging from 0 to 21.

These measures were used to describe the sample's symptom profile; the study was not designed or powered for quantitative hypothesis testing, and the primary analyses are qualitative in nature.

### 2.3 Qualitative component

A qualitative, exploratory study was conducted using thematic analysis of semi-structured 60-min interviews with patients, family members, and healthcare professionals. This was supported by secondary data review, with a focus on the US context. A structured three-step approach was applied:

#### Step 1: Data immersion

Existing assets, including medical literature summaries, key opinion leader insights, and clinical papers, were reviewed to validate the clinical pathway and inform the discussion guide for the interviews.

#### Step 2: Qualitative research

Semi-structured discussion guides were developed separately for each stakeholder group (patients, family members, and healthcare professionals), tailored to elicit perspectives relevant to each group's experience of the insomnia care pathway. Interviews were conducted by trained researchers, recorded with participant consent, and transcribed verbatim.

#### Step 3: Analysis

Thematic analysis was applied to interview transcripts following Braun and Clarke's framework (38), involving familiarization with the data, generation of initial codes, development of candidate themes, review and refinement of themes, and definition and naming of final themes. Coding was conducted by at least two researchers and discrepancies were resolved through discussion to ensure consistency. Trustworthiness of the data was addressed with reflexivity maintained by researchers keeping reflective notes throughout data collection and analysis; rich and detailed descriptions were provided to enhance transferability. Insights from all stakeholder groups were synthesized to develop a comprehensive patient journey map and identify priority areas for intervention. Where themes differed between stakeholder groups, these differences are explicitly noted in the Results section.

## 3 Results

### 3.1 Patient journey overview

**Figure 1:**
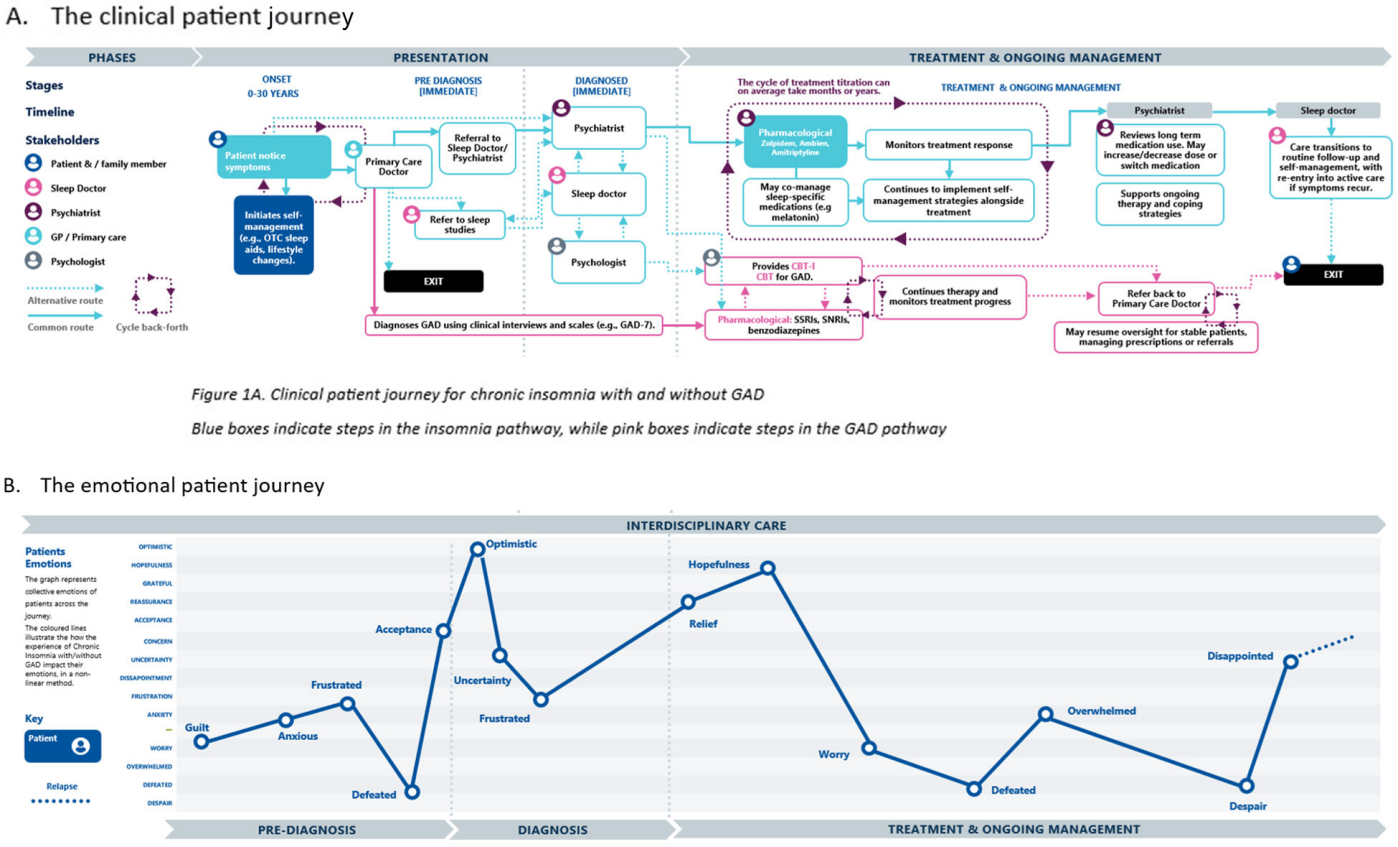
Patient clinical and emotional flow—see legend. **(A)** The clinical patient journey. **(B)** The emotional patient journey.

The patient journey for chronic insomnia, with and without comorbid GAD, was mapped across four stages: pre-diagnosis, diagnosis, and treatment and ongoing management (Figure 1).

Patients commonly delayed seeking help, often normalizing their symptoms or feeling constrained by stigma, and many relied on over-the-counter remedies for extended periods before engaging with healthcare services. Diagnostic delays were further compounded by the absence of standardized tools for early detection, and while referral to sleep centers is standard practice in the United States, attendance was inconsistent due to cost barriers and stigma. Treatment was frequently described as a trial-and-error process, with healthcare professionals acknowledging an over-reliance on pharmacological interventions despite CBT-I being the recommended first-line treatment, and limited access to non-drug options in practice.

Long-term support was reported as fragmented, with poor continuity of care and limited data sharing between mental health and sleep services. Collectively, these findings highlight a patient journey marked by delays, fragmentation, and reliance on medication, underscoring the need for integrated, patient-centered approaches to care.

### 3.2 Stage-by-stage findings

#### 3.2.1 Pre-diagnosis—delay, stigma, silence

The interviews revealed a complex and often prolonged pre-diagnosis journey shaped by differing perspectives among patients, family members, and healthcare professionals (HCP). Family members reported that they could clearly observe changes in sleep patterns and behavior yet felt helpless to intervene or support their loved ones effectively. Patient experiences were characterized by long periods of struggling in silence, self-blame, and reliance on self-management strategies before presenting to any healthcare service. Family members reported clearly observing changes in their relatives' sleep patterns and behavior, yet feeling helpless to intervene effectively. Healthcare professionals, by contrast, recognized insomnia as a serious medical condition but substantially underestimated the duration of delays patients described.

Patients themselves often normalized their symptoms, attributing them to lifestyle or personality rather than recognizing them as a medical problem. Many reported self-managing for extended periods, in some cases decades, using over-the-counter remedies, sleep hygiene strategies and calming applications. These self-management efforts were rarely successful in resolving symptoms. Stigma was a pronounced barrier, with patients reporting reluctance to seek help due to shame and a sense of personal failure:

• “*I've had trouble sleeping almost as long as I can rem*ember. *It led me to withdrew from public life and felt disconnected from my purpose”* (*patient)*

• “*Fatigue was perceived as laziness… I felt isolated in the workplace*.” (patient)

Family members described observing the gradual deterioration of their relative's sleep and wellbeing but normalizing it over time:

• “*‘I just assumed it was how she slept, going to bed late and waking up in the early hours, because it had been something that was going on for years and years.” (family member)*

• “*His sleep was fragmented, 20-minute naps followed by hours of wakefulness and he became jittery, paranoid, and uncomfortable in public settings.”* (family member)

Healthcare professionals acknowledged that patients delay presentation, typically estimating this as one to 6 months, a substantial underestimate relative to the years or decades described by patients themselves. Clinicians framed insomnia predominantly in terms of its functional and psychosocial consequences, and commonly noted it arising in the context of broader conditions such as GAD or pain. They recognized that many patients had attempted OTC medications and sleep hygiene strategies before presenting, often reaching crisis point before diagnosis:

• “*Patients will say I've always slept 4 h. They think it's part of their norm. It takes a general health screening to uncover the issues and for patients to realize it's an actual medical problem that needs intervention.” (General Practitioner)*

• “*I see insomnia routinely though mainly as part of wider issues like GAD or pain” (General Practitioner)*

Across all groups, universal recognition of the frequent co-occurrence of insomnia and GAD was evident, with anxiety described as exacerbating sleep difficulties through a loop of worry and hyperarousal, keeping patients awake and intensifying both conditions. Together, these perspectives underscore the disconnect between patient experiences, family observations, and clinical assumptions, illustrating how stigma, normalization, and fragmented pathways contribute to delayed diagnosis and unmet needs:

• “*They feel like their life is disrupted... things are going to spiral.” (General Practitioner)*

#### 3.2.2 Diagnosis—relief, validation, unmet expectations

At the point of diagnosis, patients often described a sense of relief and validation, accompanied by high expectations that their sleep problems would now be addressed. Family members shared this reassurance, comforted that the condition was formally recognized and medical intervention would follow. However, these initial hopes were frequently tempered by brief consultations, limited information provision, and delayed access to evidence-based treatment:

• “*The initial consultation with the psychiatrist was brief and not in-depth. I was offered over-the-counter options before moving to prescription medication.” (Patient)*

• *The diagnosis of chronic insomnia was not a surprise. I had always considered my sleep issues to be chronic. My main question was what could be done, and I would now get the help I really needed.” (Patient)*

In the United States, patients may be referred to a sleep center for formal polysomnographic assessment, though this did not always occur. Where referrals were made, attendance was inconsistent, driven by concerns about cost, discomfort with the assessment environment, and limited information. Patients who did attend frequently reported disappointment at not seeing a sleep consultant in person during the assessment and felt let down by the impersonal nature of the experience.

Healthcare professionals acknowledged that patients sought immediate relief and often became irritated when offered OTC options, recognizing that this could initiate another cycle of trial-and-error use. Clinicians also highlighted systemic constraints, including high patient volumes—one specialist reported seeing 300 patients per month—and limited consultation time, which restricted their ability to provide the extended support patients needed at this critical juncture:

• “*Most patients just express feeling validated that their issue is being recognized and that now that they have a diagnosis, they can potentially qualify for appropriate interventions or treatments*.” (Psychiatrist)

• “*The main barrier to confirming diagnosis is thoroughness of answering the questions and that they don't hold anything bac*k.” *(General Practitioner)*

There was no consensus among patients or providers on the link between insomnia and anxiety, with some attributing sleep difficulties to stress alone and others viewing anxiety as a separate condition. Collectively, the diagnosis stage was characterized by relief and validation, but also unmet expectations, fragmented guidance, and the beginning of a trial-and-error treatment process:

• “*Feeling validated... relieved that somebody has listened to them.”* (Psychiatrist)

• “*They're worried, they're frustrated... but once something is being done, they're less anxious.” (Sleep doctor)*

#### 3.2.3 Treatment—partial relief, medication, concerns, barriers to CBT-I

During the treatment phase, patients described partial relief in some cases but, more often, the beginning of a longer and uncertain journey toward sustainable management. Although CBT-I is the recommended first-line treatment for chronic insomnia, healthcare professionals in this study reported that pharmacological interventions were more commonly initiated in practice, largely due to time pressures in clinical settings and the need for immediate symptom relief. This gap between guideline recommendations and real-world practice was widely acknowledged:

• “*Some patients respond really well... others give up after one bad therapist experience.”* (General Practitioner regarding CBT-I)

• “*It's one of the most treatable conditions with the worst outcomes*.” (Psychiatrist)

Awareness of CBT-I among patients was limited. Several reported that CBT-I had not been offered by their healthcare provider, and some had not heard of it prior to the present study. Barriers to CBT-I implementation included limited availability of trained practitioners, high dropout rates, cost, time commitment, and concerns about patient engagement. As a result, CBT-I was frequently recommended only when patients explicitly requested non-pharmacological options or when pharmacological approaches had already failed. Despite this, patients consistently preferred non-pharmacological approaches and expressed reluctance to rely on medication due to concerns about dependency and side effects:

• “*I worry about long-term dependency and the challenge of finding effective treatments as her husband ages.”* (Family member)

• “*I try to avoid dependency on sleep medication and prefer natural methods.”* (Patient)

Concerns about medication dependency and side effects were frequently expressed by both patients and family members. Many patients described weighing the benefits of pharmacological relief against perceived risks of long-term use, and several reported tapering or avoiding medication despite experiencing continued symptoms. A strong preference for personalized treatment, collaborative decision-making, and flexible care plans was evident across the patient and family member accounts. Anxiety may compound these difficulties by making patients feel overwhelmed or hopeless when considering treatment options, further undermining engagement with longer-term behavioral approaches.

#### 3.2.4 Ongoing management—adaptation, family strain, fragmented system, need for integration

In the ongoing management stage, patients described a gradual and often difficult shift in expectations, from seeking a definitive cure to accepting insomnia as a chronic condition and focusing on strategies to maintain functionality. Over time, many developed pragmatic approaches, defining success as achieving sleep that was functional rather than perfect. This shift should be understood as clinically adaptive: in the context of CBT-I and insomnia recovery, reducing the pressure to achieve perfect sleep and accepting that some nights will be difficult is an important therapeutic mechanism. Accepting ‘good enough' sleep enables patients to disengage their attention from sleep, reduce sleep-related anxiety, and re-engage with daytime activities, an approach that supports recovery from insomnia:

• ”*I've come to accept that anything above 5.5 h of sleep is good and I can cope with that.“* (Patient)

Family members varied in their involvement, with some actively engaged in emotional and practical support while others felt uncertain about how to contribute effectively. Several reported cumulative emotional strain, particularly when symptoms persisted or worsened. The absence of dedicated resources for families and forums for shared experience contributed to emotional fatigue:

• “*I worked as a medical transcriptionist... but I realized I couldn't keep a job because of all the appointments and meetings.”* (Family member)

Healthcare professionals highlighted the fragmented nature of long-term insomnia care, particularly when patients were required to navigate multiple specialties. The absence of shared records, centralized coordination, and integrated treatment plans often led to duplication, gaps, or conflicting advice. Clinicians expressed a desire for more joined-up systems, including digital platforms and multidisciplinary teams, to support continuity and collaboration across providers:

• “*I just don't feel like there's a lot out there... I need more options.” (General Practitioner)*

• “*It depends on what the patient can afford... finances are a huge barrier.” (General Practitioner)*

## 4 Discussion

### 4.1 Summary of key insights

Findings from patient, family member, and healthcare professional interviews highlight significant psychological and systemic challenges associated with chronic insomnia, both with and without comorbid GAD. Patients described a high emotional burden characterized by frustration, hopelessness, and repeated treatment failures. A consistent theme across the patient accounts was a gradual shift toward accepting ‘good enough' sleep rather than seeking full resolution; a change that reflects not resignation, but a clinically meaningful adaptive process. In the context of CBT-I, reducing perfectionistic sleep expectations is a recognized therapeutic target that supports recovery by diminishing hyperarousal, reducing pre-sleep anxiety, and enabling re-engagement with valued activities despite imperfect sleep.

Delays in diagnosis and access to effective treatment emerged as a pervasive issue. Many patients normalized their symptoms for years, sometimes decades, and relied on self-management strategies before approaching formal healthcare services. Although healthcare professionals acknowledged the existence of delays, they consistently underestimated their duration, often citing months rather than the far longer time frames reported by patients. By the time individuals sought help, they described a strong desire for meaningful intervention rather than a repetition of introductory advice they had trialed extensively. Several participants described frustration when initial consultations focused predominantly on reiterating generic sleep hygiene guidance; perceived as a step backwards and misaligned with their readiness for targeted treatment. This mismatch between patient expectations and clinical practice contributed to feelings of dismissal and risked prolonging the pathway to evidence-based care.

Post-diagnosis, treatment options were limited in practice. Healthcare professionals described an over-reliance on pharmacological interventions, driven by time constraints and restricted access to non-pharmacological alternatives. Barriers to CBT-I were widely reported. Sleep hygiene advice, while commonly offered, is not a treatment for insomnia disorder in its own right and does not meet the standard of evidence-based care. Patients consistently described trial-and-error treatment experiences with inconsistent follow-up, and expressed a preference for personalized, flexible care plans over one-size-fits-all approaches.

The scarcity of psychological support was evident across all stakeholder groups. Family members, despite playing a key role in emotional and practical support, were often excluded from care discussions and lacked access to resources or guidance. Their accounts revealed caregiver strain, role disruption, and a desire for greater inclusion in care planning. Healthcare professionals recognized the need for integrated mental health care but reported systemic barriers to implementation that precluded the joined-up care that patients and families needed.

### 4.2 Insights in the context of existing literature

These findings align with existing literature on chronic insomnia. O'Regan et al. (6) reported similar themes in Canada and Europe, including the substantial burden experienced by patients, lack of recognition of insomnia as a medical condition, and delays in seeking medical help and diagnosis. The present research reinforces these observations in the US context, where the insurance-based healthcare system creates additional structural barriers including cost, coverage gaps, and variability in specialist availability, that compound the delays and treatment challenges already described in the literature.

The comorbidity of GAD with chronic insomnia further complicates diagnosis and treatment pathways in ways consistent with existing research on the bidirectional relationship between the two conditions. The finding that neither patients nor providers consistently recognized the causal links between insomnia and anxiety reflects a broader knowledge gap highlighted in the literature (4–6), and one with significant treatment implications: treating each condition as independent risks perpetuating the cycle of mutual exacerbation.

The finding that pharmacological treatment predominates despite CBT-I being the recommended first-line therapy is consistent with reports from other healthcare systems but is particularly pronounced in the US context, where access to CBT-I is constrained by workforce shortages, insurance coverage limitations, and system fragmentation. Healthcare professionals in this study expressed a genuine awareness of CBT-I's efficacy but described systemic conditions that made pharmacological prescribing a practical necessity rather than a clinical preference.

### 4.3 Clinical implications

The findings of this study have several important clinical implications for practitioners, healthcare systems, and policy-makers working in the area of insomnia and comorbid anxiety. CBT-I is the recommended first-line treatment for chronic insomnia, with efficacy established across multiple populations; yet medication remains the most commonly delivered intervention in practice due to systemic barriers. Considering the US healthcare context, with its insurance-based structure and variability in specialist access, addressing this gap would require investment in CBT-I training, scalable delivery models (including digital platforms), and improved insurance coverage and referral pathways. Practitioners and patients should also understand that sleep hygiene advice, while commonly offered, is not evidence-based treatment for insomnia disorder on its own. Psychoeducation about the bidirectional insomnia–anxiety relationship is important to ensure patients and providers do not treat each condition as wholly independent. Family members should be more systematically included in assessment, care planning, and ongoing management, given the caregiver burden documented here. Finally, financial barriers to care, including sleep center assessments and psychological therapy, disproportionately affect those with limited insurance coverage and require policy-level attention alongside the development of integrated care models that coordinate across primary care, sleep medicine, and mental health services.

### 4.4 Limitations

Several limitations should be considered when interpreting these findings. The qualitative design does not permit causal inferences, and findings reflect the perspectives of a specific sample of participants recruited in the United States; transferability to other healthcare contexts should be approached with caution. The sample size of 29 participants, while appropriate for qualitative inquiry, means that perspectives from groups not represented in this sample may not be captured. The study did not include participants from minority ethnic communities or from rural areas, where access barriers, and cultural factors may shape the insomnia experience differently. Recruitment methods may also have introduced self-selection bias, as participants willing to take part in research interviews may differ from those who do not engage with care at all. Additionally, the quantitative data collected (ISI and GAD-7) were used descriptively and were not designed or powered for inferential analysis; no hypothesis testing was performed, and these data should be interpreted as a characterization of the sample's symptom burden rather than as quantitative research outcomes. Finally, the cross-sectional nature of the interviews captures experiences at a single point in time and does not reflect the dynamic, evolving nature of chronic insomnia over the lifespan.

## 5 Conclusion

This research uniquely captures how the bidirectional relationship between chronic insomnia and anxiety disorders manifests in real life, drawing on the perspectives of patients, family members, and healthcare professionals. By mapping the patient journey across stages of pre-diagnosis, diagnosis, treatment, and ongoing management, the findings highlight significant unmet needs and the emotional burden carried by those affected. The complex interplay between insomnia and anxiety intensifies symptom severity, complicates treatment pathways, and exacerbates the toll on patients and their families. Across all stakeholder groups, experiences revealed delays in recognition and diagnosis, reliance on trial-and-error treatment approaches, and limited access to psychological support. Despite CBT-I being the recommended first-line treatment for chronic insomnia, it remains inaccessible to many patients due to systemic barriers. Together, these insights underscore the urgent need for integrated, patient-centered care models that address both sleep and mental health, expand access to evidence-based non-pharmacological interventions, and incorporate family perspectives into long-term management strategies. Future research should examine the effectiveness of integrated care models in the US context, explore the experiences of under-served and under-represented populations, and evaluate whether digital CBT-I delivery can close the access gap identified in this study.

## Data Availability

The original contributions presented in this study are included in the article/supplementary material, further inquiries can be directed to the corresponding authors.
